# Opossum Cathelicidins Exhibit Antimicrobial Activity Against a Broad Spectrum of Pathogens Including West Nile Virus

**DOI:** 10.3389/fimmu.2020.00347

**Published:** 2020-03-03

**Authors:** Hye-sun Cho, Joori Yum, Andy Larivière, Nicolas Lévêque, Quy Van Chanh Le, ByeongYong Ahn, Hyoim Jeon, Kwonho Hong, Nagasundarapandian Soundrarajan, Jin-Hoi Kim, Charles Bodet, Chankyu Park

**Affiliations:** ^1^Department of Stem Cell and Regenerative Biotechnology, Konkuk University, Seoul, South Korea; ^2^Laboratoire Inflammation, Tissus Epithéliaux et Cytokines, LITEC EA 4331, Université de Poitiers, Poitiers, France

**Keywords:** antimicrobial peptides, antiviral function, West Nile virus, host defense peptides, cathelicidins, *Monodelphis domestica*, gray short-tailed opossum, circular dichroism

## Abstract

This study aimed to characterize cathelicidins from the gray short-tailed opossum *in silico* and experimentally validate their antimicrobial effects against various pathogenic bacteria and West Nile virus (WNV). Genome-wide *in silico* analysis against the current genome assembly of the gray short-tailed opossum yielded 56 classical antimicrobial peptides (AMPs) from eight different families, among which 19 cathelicidins, namely ModoCath1 – 19, were analyzed *in silico* to predict their antimicrobial domains and three of which, ModoCath1, -5, and -6, were further experimentally evaluated for their antimicrobial activity, and were found to exhibit a wide spectrum of antimicroial effects against a panel of gram-positive and gram-negative bacterial strains. In addition, these peptides displayed low-to-moderate cytotoxicity in mammalian cells as well as stability in serum and various salt and pH conditions. Circular dichroism analysis of the spectra resulting from interactions between ModoCaths and lipopolysaccharides (LPS) showed formation of a helical structure, while a dual-dye membrane disruption assay and scanning electron microscopy analysis revealed that ModoCaths exerted bactericidal effects by causing membrane damage. Furthermore, ModoCath5 displayed potent antiviral activity against WNV by inhibiting viral replication, suggesting that opossum cathelicidins may serve as potentially novel antimicrobial endogenous substances of mammalian origin, considering their large number. Moreover, analysis of publicly available RNA-seq data revealed the expression of eight ModoCaths from five different tissues, suggesting that gray short-tailed opossums may be an interesting source of cathelicidins with diverse characteristics.

## Introduction

Antimicrobial peptides (AMPs), known as host defense peptides (HDPs), serve as alternatives to antibiotics owing to their antimicrobial properties and immunomodulatory responses, along with the rare possibility of acquiring bacterial resistance (1). Cathelicidins are a family of AMPs that have been identified in most vertebrates as innate immune defense peptides; they contain characteristic well-conserved cathelin-like domains (CLDs) comprising four Cys residues and a mature bioactive peptide at the C-terminal end (2). Cathelicidin propeptides undergo post-translational modification through proteolytic cleavage by specific enzymes, releasing the active antimicrobial domain (3). The number of cathelicidin genes differs among species, from one in humans to at least twelve in the opossum (4).

The identification and characterization of endogenous AMPs has been limited to only a few species. However, the availability of genome sequences from diverse species along with AMP databases and bioinformatic tools facilitates the identification of novel AMPs (5, 6).

Marsupials differ from eutherian mammals, particularly in their reproductive and developmental traits (7, 8). For instance, marsupials are exposed to harsh environments containing pathogenic bacteria during early development within a brood pouch or burrow containing an abundant and diverse microbial flora (9, 10). Cathelicidins in milk and the brood pouch lining immunologically protect naïve joeys from harmful bacteria in the environment (11, 12). Therefore, such biological characteristics may have contributed to the expansion and diversification of AMPs during evolution in marsupials (4, 13). Cathelicidins of *Monodelphis domestica* have been studied previously; however, the genes were not completely identified and characterized (14).

Although several studies have reported that LL-37 exerts antiviral activity against human immunodeficiency virus (HIV), herpes simplex virus type 1 (HSV-1), influenza A virus, and Zika virus, the list of AMPs exhibiting antiviral effects and their characterization remain limited (15–19).

West Nile virus (WNV) is an arthropod-borne virus of genus *Flavivirus*, similar to the Dengue, Zika, or yellow fever viruses. WNV has recently emerged in different regions worldwide and poses a major threat to public health (20). This mosquito-borne virus causes infections in humans and is considered the primary cause of viral encephalitis worldwide (21).

At the initial site of viral inoculation, the skin serves as a first-line host defense against flaviviruses and leads to the initiation of the early innate immune response (22). Keratinocytes, the most abundant epidermal cells, are permissive to WNV and express inflammatory and antiviral proteins upon infection (23). Thus far, no antiviral agent to combat WNV infections or vaccines are available. Therefore, the characterization of antimicrobial peptides provides insights into new antiviral and antibacterial therapies.

## Materials and Methods

### *In silico* Identification of AMP-Like Sequences From the Genome of *Monodelphis domestica*

The sequences of 1,173 non-redundant AMPs of mammalian, avian, and fish origins were downloaded from UniProtKB/Swiss-Prot^1^, using the query “antimicrobial peptide AND reviewed: yes.” Consequently, 420 sequences corresponding to eight major AMP families including alpha-defensin, apolipoprotein A2, beta-defensins, BPI/LBP superfamily, calycins, cathelicidins, hepcidin, and LEAP-2 were identified (Supplementary Table S1). Thereafter, BLASTp and tBLASTn analyses were carried out^2^ against the reference genome of the opossum (GCF_000002295.2 MonDom5). Furthermore, keyword searches were carried in the NCBI and Immunome Database for Marsupials and Monotremes (IDMM) (24) with the query, “cathelicidin AND *Monodelphis domestica*.”

### *In silico* Functional Characterization and Nomenclature of Cathelicidin-Like Sequences in the *Monodelphis domestica* Genome

Exons/introns were predicted using Splign Transcript to Genomic Alignment Tool^3^ (25). Signal peptides and CLDs were determined using SignalP 4.1 server^4^ (26) and HMMER^5^ (27), respectively. DBAASP^6^ (28) and Antimicrobial Sequence Scanning System (AMPA^7^) (29) were used to predict potential antimicrobial activity using the default threshold. Protein secondary structures were analyzed using the PSIPRED protein sequence analysis workbench^8^. Proteolytic cleavage sites were predicted to define the mature peptide region, using ExPasy PeptideCutter^9^ and PROtease Specificity Prediction servER (PROSPER^10^) (30), respectively. The mature peptide regions in the predicted cathelicidin sequences of *M. domestica* were named ModoCath 1 to 19, concurrent with the previous annotation in IDMM, where “Modo” and “Cath” stand for *M. domestica* and cathelicidin, respectively. Hydrophobicity, net charges, molecular weight and sequence similarities of ModoCaths were analyzed using APD3^11^ (31) and Protparam tool^12^.

### Bioinformatic Analysis of ModoCath Expression Using RNA-Seq Data

We downloaded 74 RNA-seq runs of *Monodelphis domestica* from NCBI SRA database^13^ (Supplementary Table S2). Expression levels of cathelicidins were determined relative to *EMC7* (accession, XM_001380762.4). Downloaded fastq sequences were mapped to the full-length coding sequences of 19 ModoCaths and *EMC7*, using BWA aligner (version 0.7.17) (32). Sorted bam files with uniquely mapped reads were obtained using samtools (version 1.9) (33). The average depth and coverage of expressed transcripts was calculated using bedtools (version 2.25.0) (34) and R software (version 3.6.0) (35).

### Peptide Synthesis and Evaluation of Antibacterial Activity

Peptides corresponding to the predicted antimicrobial regions of ModoCath1 (ΔModoCath1, N-VKRTKRGARRGL TKVLKKIFGSIVKKAVSKGV-C), ModoCath5 (ΔModoCath5, N-WYQLIRTFGNLIHQKYRKLLEAYRKLRD-C), ModoCath6 (ΔModoCath6, N-VRRSKRGIKVPSFVKKVLKDVVSESIS-C) and PMAP36 (N-GRFRRLRKKTRKRLKKIGKVLKWIPPIVGSI PLGCG-C) were synthesized via solid-phase peptide synthesis and purified via high-performance liquid chromatography using a commercial service (GenScript, Piscataway Township, NJ, United States). The MIC of synthesized peptides was determined against a panel of bacteria comprising 3 gram-positive strains, *Staphylococcus aureus* ATCC 6538 (ATCC, Manassas, VA, United States), *Bacillus cereus* ATCC 10876, and *Enterococcus faecalis* ATCC 29212, and 3 gram-negative strains, *Escherichia coli* ATCC 25922, *Pseudomonas aeruginosa* ATCC 27853, and *Salmonella typhimurium* ATCC 14028. Ampicillin (Sigma Aldrich, St. Louis, MO, United States) and gentamicin sulfate (Sigma Aldrich) were used as controls for antimicrobial activity. The MIC was determined using a colorimetric method specified by the Microbial Viability Assay Kit-WST (Dojindo, Kumamoto, Japan) in accordance with the manufacturer’s protocol and the Clinical and Laboratory Standards Institute (CLSI) guidelines (2018). Briefly, four colonies of each bacterium were inoculated into 5 mL Luria-Bertani (LB) broth medium at 37°C for 4 to 6 h. The cells were washed with sterile saline (0.9% NaCl) twice and seeded in a single well of a 96-well plate at the cell density of 10^5^ CFU/well. Subsequently, 180 μL/well of fresh Mueller-Hinton broth (MHB) was added to the plate. Different concentrations of each peptide and reference antibiotics were serially diluted in 10 μL of MHB and added to each well. The plate was incubated at 37°C for 6 h. Cation-adjusted MH broth (CAMHB) was used to culture *E. faecalis*. Subsequently, 10 μL of the coloring reagent was added, and cells were incubated at 37°C for 2 h. UV absorbance was measured for each well at 450 nm, using a microplate spectrophotometer (xMark spectrophotometer; Bio-Rad, Hercules, CA, United States). MIC values were determined when the difference in the optical density (OD) between treatments and blanks (media and coloring reagent only) decreased to < 0.05. Experiments were conducted in triplicate. The MIC of ΔModoCath1 and 5 in different physiological salts (150 mM NaCl, 1 mM MgCl_2_, 4.5 mM KCl and 2.5 mM CaCl_2_) and pH conditions (pH 5, 6, and 7) was also determined against *E. coli* (ATCC 25922). The pH conditions were achieved by using acetic acid (Sigma Aldrich).

### *In vitro* Analysis of Serum Stability of the AMPs

The Ethics Committee of the Konkuk University Hospital approved the use of human serum samples for research studies, and human serum was obtained from Konkuk University Medical Center (KUMC) Biobank.

Antimicrobial peptides were dissolved in 25% (v/v) pooled human serum from five individuals, and incubated at 37°C. Aliquots were extracted in triplicate after 0, 60, and 120 min incubation, and their antimicrobial activity against *E. coli* (ATCC 25922) was assessed using the incubated samples, as described above.

### Determination of *in vitro* Mammalian Cell Cytotoxicity

Two mammalian cell lines, including human embryonic kidney cells (HEK293T) and human breast cancer cells (MCF7) were cultured in Dulbecco’s modified Eagle’s medium (DMEM; Hyclone^TM^, Logan, UT, United States) supplemented with 10% FBS (Hyclone^TM^) and 1% penicillin/streptomycin (Hyclone^TM^) and incubated at 37°C and 5% CO_2_ up to 80% confluence. Cellular adherence to the substratum was disrupted using Accutase (Innovative Cell Technologies, San Diego, CA, United States). In total, 1 × 10^4^ to 4 × 10^4^ cells in each well of a 96-well plate containing 8, 16, 32, and 64 μg/mL of ModoCath peptides were incubated for 24 h at 37°C and 5% CO_2_. Additionally, HEK293T cells were incubated in the FBS-free medium. Triton X 100 (Sigma Aldrich) was used as a positive control for complete cell lysis, and untreated cells were used as the negative control. After incubation, the medium was removed from the wells, and 10 μL of coloring solution (Cell Proliferation Reagent WST-1^TM^; Sigma Aldrich) and 100 μL of DMEM (Hyclone^TM^) were added to the wells in accordance with the manufacturer’s protocol. Absorbance was measured for each well at 440 nm (peptide-treated and control) and 650 nm (background) and recorded as the OD, using a microplate reader (xMark^TM^ spectrophotometer; Bio-Rad). Cell viability was calculated using the following equation:

(1) $$CellViability\left(\%\right)=100\times \frac{\left(O\,D\,p\,e\,p\,t\,i\,d\,e-O\,D\,b\,a\,c\,k\,g\,r\,o\,u\,n\,d\right)}{\left(O\,D\,n\,e\,g\,a\,t\,i\,v\,e-O\,D\,b\,a\,c\,k\,g\,r\,o\,u\,n\,d\right)}$$

All experiments were performed in triplicate.

### Circular Dichroism Spectroscopy

Circular dichroism (CD) spectra signals were recorded at 25°C using a Jasco J-810 spectropolarimeter (Jasco, MD, United States) at an emission range of 195 – 260 nm, scanning speed of 50 nm/min, 1 nm bandwidth, 4 s response time, and four accumulations using a rectangular quartz cell (0.1 cm path length). All peptides were scanned at a concentration of 25 μM dissolved in 10 mM sodium phosphate buffer, pH 7.0. Lipopolysaccharide (LPS, Sigma Aldrich) titrations were carried out with increasing concentrations from 0 – 0.16 mg/mL to 25 μM peptide in 10 mM sodium phosphate buffer pH 7.0. LPS was prepared via temperature cycling between 4 and 70°C, and vortexed for 10 min. LPS was stored at 4°C overnight before use. The CD spectrum signal for the peptides was obtained after subtracting LPS respective spectrum from that of LPS and peptide mixtures. All experiments were triplicated.

### Dual-Dye Membrane Disruption Assay

Four colonies of *B. cereus* (ATCC 10876), *E. coli* (ATCC 25922), and *S. aureus* (ATCC 6538) were inoculated into 5 mL LB broth and incubated at 37°C for 5 h. Cells suspensions were centrifuged at 3000 × g and 25°C, washed and resuspended in phosphate buffered saline (PBS) + (0.14 M NaCl, 2.7 mM KCl, 10 mM Na_2_HPO_4_, and 1.8 mM KH_2_PO_4_ supplemented with 10 mM glucose and 0.5 mM MgCl_2_; pH 7.4) to an OD_600_ of 0.1. Concurrently, a blank PBS+ sample without cells and cells treated with Nisin (Sigma Aldrich) and vancomycin (Sigma Aldrich) were used as controls (36). TO-PRO-3 iodide (Sigma Aldrich) and DiOC_2_(3) (Sigma Aldrich) dyes were then added into the sample and controls to a final concentration of 625 nM and 10 μM, respectively. The plates were incubated at 25°C in the dark for 5 min, and the cells were then treated with ModoCath peptides to final concentrations of 0.1×, 0.2×, and 1× MIC, respectively. Thereafter, the absorbance spectra for TO-PRO-3 iodide and DiOC_2_ were determined (Supplementary Figure S1). The absorbance of the plates was read at λ_ex_ 640 nm and λ_em_ 700 nm for TO-PRO-3 iodide and λ_ex_ 480 nm and λ_em_ 530 nm for DiOC_2_(3) using a fluorescence microplate reader (Gemini EM, Molecular Devices, Sunnyvale, CA, United States), where λ_ex_ and λ_em_ indicate wavelengths for excitation (λ_ex_) and emission, respectively.

### Field Emission Scanning Electron Microscopy

*Escherichia coli* (ATCC 25922) cells at an OD_600_ value of 0.2 were inoculated in LB medium with 1.5 μg/mL ΔModoCath1, 10 μg/mL ΔModoCath5 or 4 μg/mL PMAP36 followed by incubation for 2 and 4 h, respectively, at 37°C. The bacterial cells were harvested by centrifugation at 4,500 rpm, after which the pellets were washed twice with PBS and fixed with 2.5% glutaraldehyde (Sigma Aldrich) in PBS for overnight at 4°C. Cells were then washed thrice with PBS and dehydrated using graded ethanol at 50, 70, and 90% for 10 min each, and 100% for 15 min. Subsequently, samples were dried with hexamethyldisilazane (Daejung Chemicals and Metals Co. Ltd., Siheung, South Korea) for 15 min. For observation, prepared samples were sputter-coated with platinum using Ion Sputter MC1000 (Hitachi High-Technologies, Tokyo, Japan) prior to imaging with a Hitachi HR FE-SEM SU8010 (Hitachi High-Technologies).

### Isolation and Culturing of Normal Human Epidermal Keratinocytes From Skin Samples

The Ethics Committee of the Poitiers Hospital approved the use of human skin samples for research studies. All subjects provided written informed consent in accordance with the tenets of the Declaration of Helsinki. Normal abdominal or breast skin samples were obtained from patients undergoing plastic surgery and thoroughly washed with PBS free of calcium and magnesium (PBS; Gibco, Thermo Fisher Scientific, Waltham, MA, United States) after fat removal. The skin samples were minced into fragments of approximately 125 mm^2^, using scalpel blades. Skin samples were incubated overnight at 4°C in a dispase solution (25 U/mL; Life Technologies, Carlsbad, CA, United States). Epidermal sheets were removed from the dermis, and keratinocytes were dissociated via trypsin digestion (trypsin-EDTA; Gibco) for 15 min at 37°C. The cell suspension was then filtered through a 280-μm sterile filter. DMEM (Gibco) supplemented with 10% (vol/vol) of FBS (Gibco) was added, and the suspension was centrifuged at 300 × *g* and 25°C for 10 min. Keratinocytes were seeded at a density of 10^7^ cells in 75-cm^2^ tissue culture flask in keratinocyte-serum free medium (K-SFM; Invitrogen, Carlsbad, CA, United States) supplemented with bovine pituitary extract (25 μg/mL; Invitrogen) and recombinant epidermal growth factor (EGF) (0.25 ng/mL; Invitrogen). The cultures were incubated at 37°C in a humidified atmosphere with 5% CO_2_ until 80% confluence and then stored frozen in liquid nitrogen until use. Finally, keratinocytes were seeded in sterile 24-well culture plates at 10^5^ cells/well in K-SFM supplemented with bovine pituitary extract and EGF and cultured to 80% confluence. Cells were then starved overnight in K-SFM alone before stimulation.

### Assessment of the Viability of Keratinocytes

Primary keratinocytes were cultured in 96-well plates at 4 × 10^4^ cells per well in 0.1 mL K-SFM (Invitrogen) up to 80% confluence before being treated with various concentrations of ModoCath peptides for 24 h. Cell viability was assessed using the cell proliferation kit II (XTT; Roche, Basel, Switzerland) in accordance with the manufacturer’s protocol. The XTT labeling mixture was added after 24 h of incubation in the absence or presence of peptides at the indicated concentrations.

### WNV Strain Production

A lineage WNV clinical strain, isolated from a human brain during an epidemic occurring in Tunisia in 1997, was provided by Dr. I. Leparc Goffart (French National Reference Center for Arboviruses, Marseille, France). The viral stock was produced on the *Aedes albopictus* clone C6/36 cells (ATCC^®^ CRL-1660^TM^). Cells were cultivated in Leibovitz’s L-15 medium (Gibco) supplemented with 2% of tryptose-phosphate (Gibco) and 5% of FBS (Gibco) in a 75-cm^2^ tissue culture flask at 28°C until 50% confluence and then infected at a multiplicity of infection (MOI) of 0.01 for 72 h. Cell supernatants of infected cells and uninfected C6/36 cells, used as the control, were clarified via centrifugation in 50-mL tubes for 15 min at 1500 × *g*. Thereafter, the viral suspension and the supernatant from the uninfected C6/36 suspension were ultrafiltered using Amicon ultra-4 centrifugal filter units 100 kDa (Dominique Dutscher, Brumath, France) for 5 min at 3,000 × *g*. The viral suspension and the supernatant from the uninfected C6/36 suspension were frozen at −80°C in cryotubes containing 500 μL of Leibovitz’s L-15 medium supplemented with 0.5 M sucrose and 50 mM HEPES. The final viral titer was 10^7.97^ TCID50 (50% tissue culture infective dose) per milliliter as determined using 10-fold serial dilutions of the virus sample on Vero cell monolayers (described below).

### Viral Quantification via the End-Point Dilution Assay

Vero cells were seeded in 96-well plates the day before titration at 2 × 10^3^ cells/well in DMEM (Gibco) supplemented with 2% SVF. The suspension was successively diluted from a dilution of 10^–1^ to 10^–9^ in DMEM medium supplemented with 2% SVF. Thereafter, 100 μL of each dilution was deposited in a row of six wells. Initial data were obtained after 120 h of incubation at 37°C in 5% CO_2_. The wells containing cells with cytopathic effects were considered positive for viral infection. The titer of the viral suspension was then determined using the Kärber’s method for assessing the TCID50.

### Viral Infection

Human primary keratinocyte cultures (60–80% confluence) from three different patients were infected at a MOI of 0.1 and incubated for 24 h at 37°C in 5% CO_2_ in K-SFM (Invitrogen) medium. Cell culture supernatants and cell monolayers were harvested for viral quantification via RT-qPCR and transcriptomic analysis of inflammatory markers, as described below.

### Antiviral Assays

The antiviral properties of ModoCath peptides were first assessed by evaluating their impact on growth kinetics of the virus inoculated on primary human keratinocytes. Keratinocyte cultures from three different patients were incubated with one of the three peptides at a final concentration of 16 μg/mL for 1 h before addition of WNV at a MOI of 0.1 TCID50 per cell. Uninfected cultures with or without the peptides were used as the control. After 24 h of incubation at 37°C in 5% CO_2_ in K-SFM (Invitrogen) medium, cell culture supernatants and cell monolayers were harvested for viral quantification via RT-qPCR and transcriptomic analysis of inflammatory markers. The virucidal properties of ModoCath peptides directly on the virus were assessed by pre-incubating 0.1 mL of the virus stock (described above) with peptides for 1 h at 37°C before titration via the end-point dilution assay using Vero cells, as described above. The viral titer thus determined was compared to that of similarly assayed untreated viral suspensions.

### RNA Extraction

For viral RNA quantification in cell supernatants, 200 μL of total DNA/RNA from keratinocyte supernatants was extracted using a NucliSENS easyMAG^®^ automated system (bioMérieux, Marcy-l’Étoile, France) in accordance with the manufacturer’s protocol. For intracellular viral RNA quantification and evaluation of the host inflammatory response, total RNA was extracted from the keratinocyte monolayer using the Nucleo-Spin RNA extraction kit in accordance with the manufacturer’s instructions (Macherey-Nagel, Düren, Germany). RNA concentrations and purity were determined using the Nanodrop 2000 spectrophotometer (Thermo Fisher Scientific).

### Viral Quantification via RT-qPCR

Viral quantification in cell supernatants and keratinocytes was performed using a previously described one-step real time RT-PCR assay (23). Total RNA (5 μL) was added to the reaction mixture containing 12.5 μL of Master Mix (Invitrogen), 0.5 μL (0.2 μM) of forward (5′-GTGCGGTCTACGATCAGTTT-3′) and reverse primers (5′-CACTAAGGTCCACACCATTCTC-3′), 0.25 μL (0.1 μM) of 5′FAM and 3′Dark Quencher probe (5′-AATGTGGGAAGCAGTGAAGGACGA-3′), 0.5 μL of SuperScript III reverse transcriptase (Invitrogen) and DNA polymerase platinum Taq (Invitrogen), 0.5 μL of RNase out (Invitrogen), and 5.25 μL of water. The calibration range was determined using a transcript produced using a plasmid containing the WNV genome without the genes encoding structural proteins. Transcripts were diluted to obtain a calibration range allowing for the quantification of viral load from 10^2^ to 10^7^ RNA copies/mL.

### Transcriptomic Analysis of the Innate Antiviral Immune Response in Keratinocytes

Total RNA (1 μg) was reverse-transcribed using SuperScript II kit (Invitrogen). Quantitative real time PCR was performed in 96-well plates with a LightCycler 480 system (Roche). A reaction mixture comprised 5 μL of AceQ SYBR Green qPCR Master Mix (Vazyme Biotech, Nanjing, China), 1 μM forward and reverse primers designed using Primer 3 software, and 12.5 ng of cDNA template in a total volume of 10 μL. PCR conditions were as follows: 5 min at 95°C, 40 amplification cycles for 20 s at 95°C, 15 s at 64°C, and 20 s at 72°C. Relative mRNA expression of target genes was normalized to that of two independent control housekeeping genes (GAPDH and 28S rRNA gene) and reported using the ΔΔCT method as fold-changes in RNA: 2ΔΔ CT = 2^ΔCTsample–ΔCTreference^.

### Quantification of Type III Interferon Secretion

Keratinocyte secretion of active type III IFNs (IL-28A, IL-28B, and IL-29) was quantified using HEK-Blue^TM^ IFN-λ reporter cells expressing an inducible secreted embryonic alkaline phosphatase (InvivoGen, San Diego, CA, United States) according to the manufacturer’s instructions. The activity of the secreted alkaline phosphatase was measured as a colorimetric reaction at 630 nm using the Quanti-Blue reagent (InvivoGen).

## Results

### Identification of 56 AMP Genes From *in silico* Analysis of the *Monodelphis domestica* Genome

The strategy for the *in silico* identification of AMPs from the *Monodelphis domestica* genome is described in Supplementary Figure S2. We identified a total of 56 putative AMP genes in *M. domestica*, including one alpha-defensin, one apolipoprotein A2, 3 beta-defensins, 21 BPI/LBP superfamily members, 7 calycins, 21 cathelicidin-like, one hepcidin, and one LEAP-2 (*e*-value < 0.001; Supplementary Tables S3, S4). Among the 21 cathelicidins, 7 were previously undescribed, and their putative names were assigned as ModoCath 13 to 19 after excluding secreted phosphoprotein 24 and cathelicidin-related peptide Oh-Cath-like isoform X2, which failed to meet the characteristics of functional cathelicidins (Supplementary Tables S5, S6). The conservation of the CLD and cysteine motif among the 20 opossum cathelicidins is shown in Supplementary Figure S3. Basic proline-rich protein-like isoform X1 was excluded because it had a longer sequence than that of others.

### *In silico* Prediction of Eight *Monodelphis domestica* Cathelicidins With Antimicrobial Activity

The 21 cathelicidin-like sequences were analyzed *in silico* to predict protein secondary structures and the antimicrobial activity core region using AMPA, DBAASP and PSIPRED protein sequence analysis workbench databases. The analysis identified eight sequences, including ΔModoCath1, 2, 4 to 7, 12, and 19, which were strongly predicted to possess antimicrobial activity-related structures (Supplementary Figure S4 and Supplementary Table S6). Biochemical features of the core sequences from the eight cathelicidins, deduced using APD3 and Protparam, showed the antimicrobial activity-conferring regions to be 27 to 41 amino acids long and 3.04–4.85 kDa in molecular weight (Table 1). The ratios of hydrophobic residues and net charges for the core regions were 25 to 41% and +4 to +12, respectively. Their sequence similarities to known AMPs were less than 50%, indicating that they were novel. Interestingly, ΔModoCath12 and ΔModoCath19 shared the same core sequence (Supplementary Figure S4 and Table 1).

**TABLE 1:** Characteristics of the antimicrobial activity domain for the eight cathelicidins of *Monodelphis domestica* selected for their antimicrobial activity.

Name in this study	Core sequences with antimicrobial activity	Length	<H>^a^	*z*^b^ (+)	Molecular weight (Da)	Similarity (%)^c^
ΔModoCath1	VKRTKRGARRGLTKVLKKIFGSIVKKAVSKGV	32	37	12	3510.37	41.66
ΔModoCath2	VKRTKRGIKKGISKVLKKFFSSMIKKAVSK	30	36	12	3422.31	39.47
ΔModoCath4	GIRGFWNGFRGR	12	33	3	1422.61	46.15
ΔModoCath5	WYQLIRTFGNLIHQKYRKLLEAYRKLRD	28	35	5	3623.27	35.71
ΔModoCath6	VRRSKRGIKVPSFVKKVLKDVVSESIS	27	37	6	3042.66	36.66
ΔModoCath7	IVRRSKRGIKVPGFVKKFLKDVVSETI	27	40	6	3100.79	37.50
ΔModoCath12^d^	VKRTKREISKILEEIFSTVIKIFIPKGFYKGIQLVNEIIKE	41	41	4	4849.87	38.63
ΔModoCath19^d^	VKRTKREISKILEEIFSTVIKIFIPKGFYKGIQLVNEIIKE	41	41	4	4849.87	38.63

*^*a*^The ratio of hydrophobic residues. ^*b*^The charge. ^*c*^Similarity to known AMPs. ^*d*^The sequence of the predicted antimicrobial activity domain is identical.*

### Difference in Antibacterial Specificity of ΔModoCath1, 5, and 6

Among the eight ModoCath peptides with predicted antimicrobial activity, we chemically synthesized three peptides, ΔModoCath1, 5, and 6, based on the uniqueness of their sequences which are not based on statistical evaluation (Supplementary Figures S4, S5). The antimicrobial activity of the peptides was evaluated against our bacterial panel, comprising 3 gram-negative strains, *Escherichia coli*, *Pseudomonas aeruginosa*, and *Salmonella typhimurium*, and 3 gram-positive strains, *Staphylococcus aureus*, *Bacillus cereus*, and *Enterococcus faecalis*. All three peptides showed strong antibacterial activities with differences in bacterial strain specificity (Table 2). ΔModoCath1 showed the strongest and broadest activity against both gram-positive and gram-negative bacteria, with MICs of 0.75 to 3 μg/mL, except for *B. cereus* (30 μg/mL). ΔModoCath5 showed antibacterial activity toward gram-positive strains with MICs of 1.5 to 6 μg/mL. ΔModoCath6 showed bactericidal activity only against *E. coli* in our panel.

**TABLE 2:** Antimicrobial activity of three opossum cathelicidins comparing to conventional antibiotics against standard bacterial strains.

Strains	MIC (μg/mL, μM)						
	ΔModoCath1	ΔModoCath5	ΔModoCath6	Ampicillin^a^	Gentamycin^a^	Nisin^b^	Vancomycin^b^
**Gram-negative bacteria**							
*E. coli* ATCC 25922	0.75 (0.21)	5 (1.38)	5 (1.64)	1 (2.86)	1 (2.09)	> 32(9.54)	32 (22.08)
*P. aeruginosa* ATCC 27853	2 (0.57)	> 32(8.83)	> 32(10.51)	> 128(366.33)	1 (2.09)	ND	ND
*S. typhimurium* ATCC 14028	2 (0.57)	> 32(8.83)	> 32(10.51)	1 (2.86)	1 (2.09)	ND	ND
**Gram-positive bacteria**							
*S. aureus* ATCC 6538	3 (0.85)	1.5 (0.41)	> 32(10.51)	1 (2.86)	1 (2.09)	15 (4.47)	5 (3.45)
*B. cereus* ATCC 10876	30 (8.5)	6 (1.66)	> 32(10.51)	16 (45.79)	1 (2.09)	32 (9.54)	4 (2.76)
*E. faecalis* ATCC 29212	2 (0.57)	2 (0.55)	> 32(10.51)	2 (5.72)	5 (10.45)	ND	ND

*^*a*^Antibiotics for control according to the CLSI standard. ^*b*^Antimicrobials for control used in the membrane activity assay. ND, Not determined.*

### Stability of the Bactericidal Activity of ΔModoCath1 and 5 in Serum and Various Salt and pH Conditions

For the pharmaceutical application of AMPs, their stability in serum as well as the physiological condition in which they are placed are important factors to consider. We evaluated the effect of serum on the antimicrobial activity of ΔModoCath1 and 5 at different concentrations. The peptides were incubated in 25% human serum for 0–120 min, and the antimicrobial activity of the peptides against *E. coli* was assessed over time (Supplementary Figure S6). Results show that the antimicrobial activity of ΔModoCath1 and 5 was affected to varying degrees at different concentrations and incubation times. However, the activity was unaffected at >∼2× MIC after 60 min incubation, and decreased following 120 min incubation with human serum. Therefore, our results showed that the two cathelicidins, ΔModoCath1 and 5, with broad-spectrum antimicrobial activity do not exhibit significant susceptibility to inhibitory substances within human serum.

We also evaluated the antimicrobial activity of ΔModoCath1 and 5 in various salt and pH conditions (Supplementary Table S7). No inhibitory effect was observed in their activity at 150 mM NaCl, 1 mM MgCl_2_, 4.5 mM KCl or 2.5 mM CaCl_2_, all of which correspond to various physiological conditions (37). Interestingly, the MIC of ΔModoCath1 was found to decrease slightly from 0.75 to 0.5 μg/mL, potentiating the activity in the physiological salt conditions than in bacterial culture media. Regarding the varying pH conditions, a minimum of 2-fold increases were observed in the activity of ΔModoCath1 and 5 in acidic conditions (pH 5 and 6) compared to neutral pH.

### Low-to-Moderate Level Cytotoxicity of ΔModoCath1, 5, and 6 to Mammalian Cells

Cathelicidins with strong antimicrobial activity could negatively affect mammalian cells (38, 39). Therefore, the degree of cellular damage caused by ModoCaths to HEK293T and MCF7 cells and human primary keratinocytes was assessed by evaluating the viability of cells exposed to various concentrations (8–64 μg/mL) of ΔModoCath1, 5, and 6 (Table 3). Cell survivability was >∼90% at concentrations below 16 μg/mL for all tested cells for ΔModoCath1 and 6, indicating that the cells were minimally affected at that concentration. However, ΔModoCath5 showed variation in cell viability from 67 to 96% at the same concentration, showing slightly higher cytotoxicity than the other ModoCaths. Moreover, the viability of MCF7, a human breast cancer cell line, was not significantly affected by the three ModoCaths, indicating the lack of direct antitumorigenic activity (Table 3). In addition, the cytotoxicity of ModoCaths to HEK293T cells in the absence and presence of serum was not different significantly (Supplementary Table S8).

**TABLE 3:** Viability of human cells treated with varying concentrations of three opossum cathelicidins.

Peptide	Concentration (μg / mL)	Cell viability ± SD (%)		
		HEK293T	Human primary keratinocytes	MCF7
ΔModoCath1	8	108.1 ± 9.9	97.1 ± 2.9	91.2 ± 2.5
	16	105.5 ± 6.9	91.4 ± 1.6	89.2 ± 0.6
	32	66.9 ± 5.0	56.2 ± 8.4	79.1 ± 1.8
	64	55.7 ± 6.2	5.8 ± 3.7	42.4 ± 2.1

ΔModoCath5	8	93.7 ± 8.3	98.1 ± 0.6	98.4 ± 4.2
	16	67.4 ± 3.1	96.2 ± 3.5	84.6 ± 0.6
	32	47.8 ± 2.7	79.1 ± 13.0	28.4 ± 0.7
	64	35.0 ± 0.8	48.9 ± 15.5	10.3 ± 2.8

ΔModoCath6	8	103.8 ± 5.0	98.0 ± 1.6	89.2 ± 1.4
	16	95.6 ± 8.4	100.3 ± 1.3	93.2 ± 0.9
	32	89.0 ± 13.1	101.0 ± 2.6	92.8 ± 1.0
	64	88.8 ± 8.1	89.2 ± 7.2	95.2 ± 1.0

Triton X-100^a^		11.6 ± 2.5	8.2 ± 0.4	11.4 ± 0.4

*^*a*^The control for cell lysis. The experiment was triplicated.*

### Secondary Structure Formation of ΔModoCath1 and 5 After Interaction With *E. coli* LPS

**FIGURE 1:**
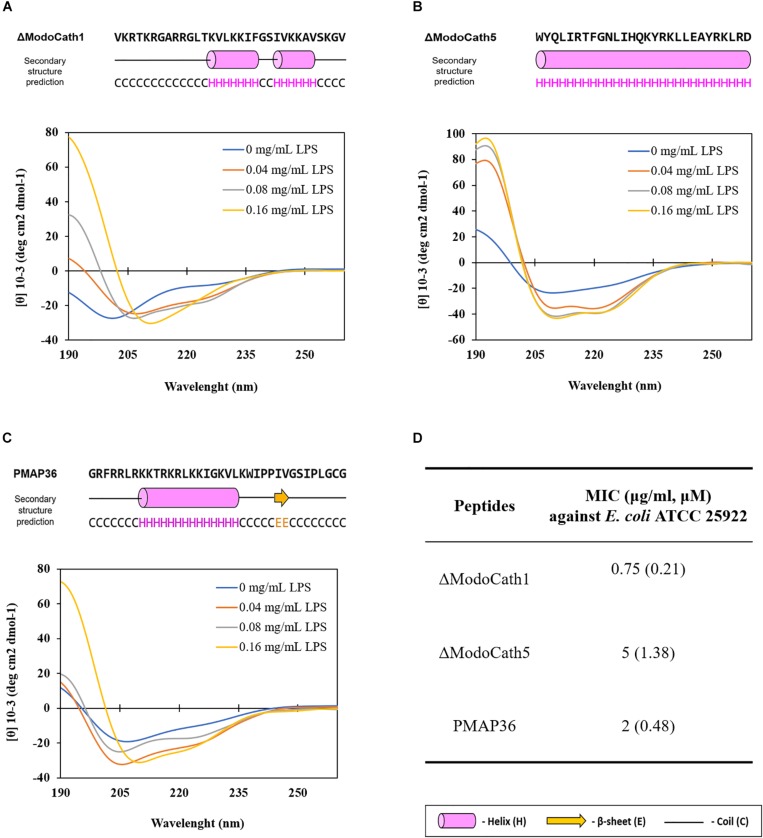
Changes in the structural conformation of ΔModoCath1 and 5 peptides upon interaction with LPS. The CD spectra were recorded for ΔModoCath1 **(A)**, Δ ModoCath5 **(B)**, and PMAP36 **(C)** following incubation with increasing concentration of LPS. The CD spectra of LPS were subtracted from their respective spectra in combination with the peptide mixtures. The predicted secondary structures of these peptides using PSIPRED are presented together with amino acid sequences. The MICs of ΔModoCath1, 5 and PMAP36 against *E. coli* are also presented **(D)**. Representative data from one set of experiments are shown.

Next, we analyzed the peptide conformation of ΔModoCath1 and 5 using circular dichroism (CD) analysis. LPS was titrated against each peptide to evaluate the secondary structure formation upon interactions. Results reveal gradual formation of the α-helical structure upon increasing concentration of *E. coli* LPS (Figure 1), demonstrating that peptide binding to LPS triggers formation of ΔModoCath1 and 5 secondary structure. Further, ΔModoCath1 contained a randomly coiled structure in LPS-free aqueous solution with a negative peak at 198 nm (Figure 1A). Surprisingly, ΔModoCath5 showed a helical structure even in aqueous solution, with a negative peak at 208 nm (Figure 1B). Although both ΔModoCath1 and 5 demonstrated increased mean residual ellipticity at 208 and 222 nm with increasing LPS concentrations and increased helicity (40), the spectra for ΔModoCath5 contained double minima at 208 and 222 nm, indicating higher helicity compared to that of ΔModoCath1. Comparatively, PMAP36, a well-characterized cathelidicin with a helical structure (41), also showed a similar CD spectral signature to ΔModoCath1 with different concentrations of LPS (Figure 1C).

### Disruption of Bacterial Membrane Permeability by ModoCaths

**FIGURE 2:**
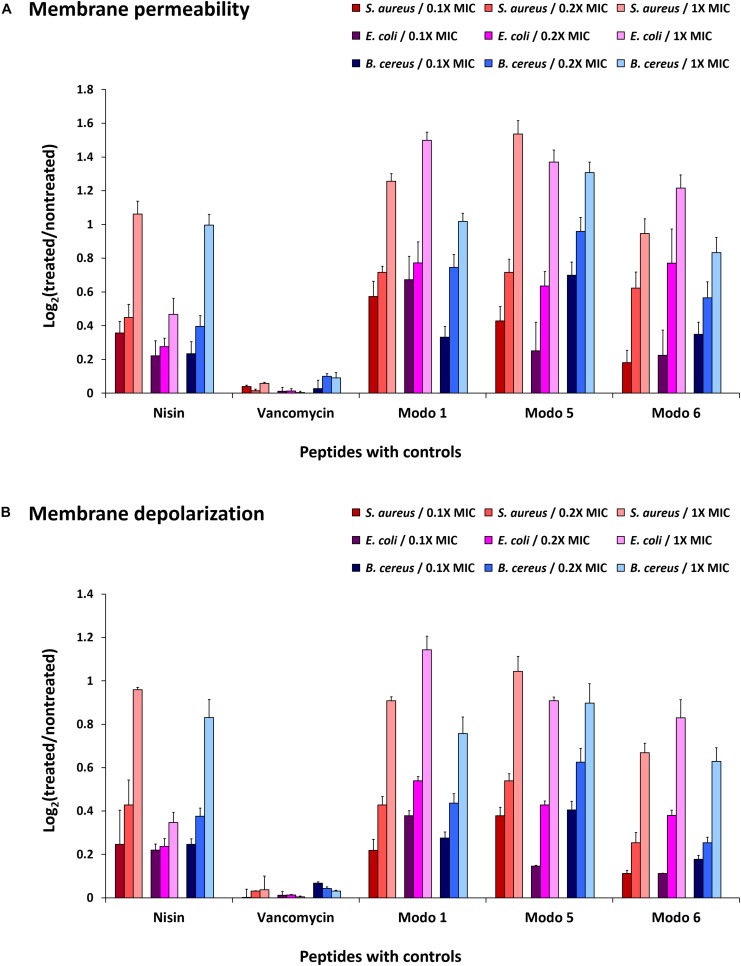
Disruption of bacterial membrane permeability by ΔModoCath peptides. The fluorescence of TO-PRO-3 iodide and DiOC_2_(3) was detected from *B. cereus* (ATCC 10876), *E. coli* (ATCC 25922), and *S. aureus* (ATCC 6538) treated with ΔModoCath1, 5, and 6 at the concentration of 0.1×, 0.2×, and 1× MIC. Nisin and vancomycin were used as controls. **(A)** The fluorescence of TO-PRO-3 iodide was measured for membrane permeability at λ_ex_ 640 nm and λ_em_ 700 nm. **(B)** The fluorescence of DiOC_2_(3) was detected at λ_ex_ 480 nm and λ_em_ 530 nm for cell polarity. The log2 ratio of detected signals between treated and non-treated wells is expressed on the *Y*-axis. Error bars represent the standard deviation from three replicated experiments. Modo 1, 5, and 6 indicate ΔModoCath1, 5, and 6, respectively.

Fluorescent dyes have been used to determine the integrity of membranes (36). The increased fluorescence intensity of TO-PRO-3 iodide and DiOC_2_(3) in cells indicates the penetration of dyes to the cytoplasm due to the damaged membrane and disruption of membrane potential. The treatment of *B. cereus*, *E. coli*, and *S. aureus* with ΔModoCath1, 5, and 6 resulted in an increase in fluorescence from the cells cultured with either dyes regardless of cell type (Figure 2). This result is identical to that obtained on treatment with Nisin, a lantibiotic, known to create a pore that disrupts membrane permeability. The lower effect of Nisin treatment on the membrane permeability of *E. coli* than that of *S. aureus* and *B. cereus* was also consistent with the activity preference of Nisin on gram-positive bacteria rather than gram-negative bacteria (42).

The disruption of membrane potential, as indicated by DiOC_2_(3) fluorescence after peptide treatment, differed among bacterial strains. However, the magnitude of changes was consistent with the strength of the bactericidal activity of ModoCaths (Table 2). In contrast, vancomycin, which does not disrupt the cell membrane, showed no changes in the fluorescence output of either dye. Therefore, we concluded that the antibacterial activity of the three ModoCaths validated in this study was mediated by the increase in membrane permeability owing to membrane disruption.

### Damage to Bacterial Cell Envelop and Leakage of Cell Contents Following Exposure to ModoCaths

**FIGURE 3:**
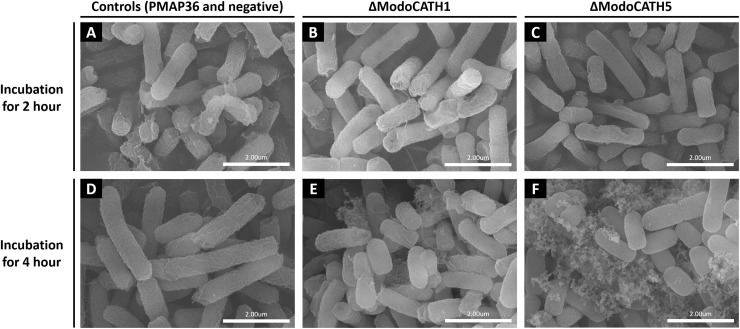
Scanning electron microscopy images of bacterial cells treated with ΔModoCath1, 5 and PMAP36. Electron micrographs showing *E. coli* cells treated with 2× MIC of ΔModoCath1 (1.5 μg/mL, **B,E**), ΔModoCath5 (10 μg/mL, **C,F**), and PMAP36 (4 μg/mL, **A**) for 2 or 4 h. Panel **D** represents the untreated control. PMAP36 was used as a representative control for membrane disrupting cathelicidins. Scale bar, 2 μm.

To visualize the morphological changes occurring in bacteria following treatment with ΔModoCath1 and 5, high resolution electron microcopy was performed. The synthesized PMAP36 peptide, which is a representative of cathelicidins with membrane disrupting activity, was used as a comparison (Figure 3A). Intact *E. coli* without any treatment was used as negative control (Figure 3D). Field emission scanning electron microscopy (FE-SEM) images of *E. coli* cells following 2 or 4h of peptide treatments showed complete or partial disruption of bacterial membranes with subsequent outflow of cytoplasm (Figures 3B,C,E,F). The images show that both ΔModoCath1 and 5 induce formation of pores (Figures 3B,C) and blisters (Figures 3E,F) on the bacterial surface, leading to leakage of cytoplasmic materials (Figures 3E,F) in peptide-exposed cells, which ultimately becomes so severe as to cause formation of coral reef-like structures among bacterial cells, most notably in *E. coli* cells treated for 4 h with ΔModoCath5 (Figure 3F).

### Strong Inhibition of West Nile Virus Replication by ΔModoCath5

**FIGURE 4:**
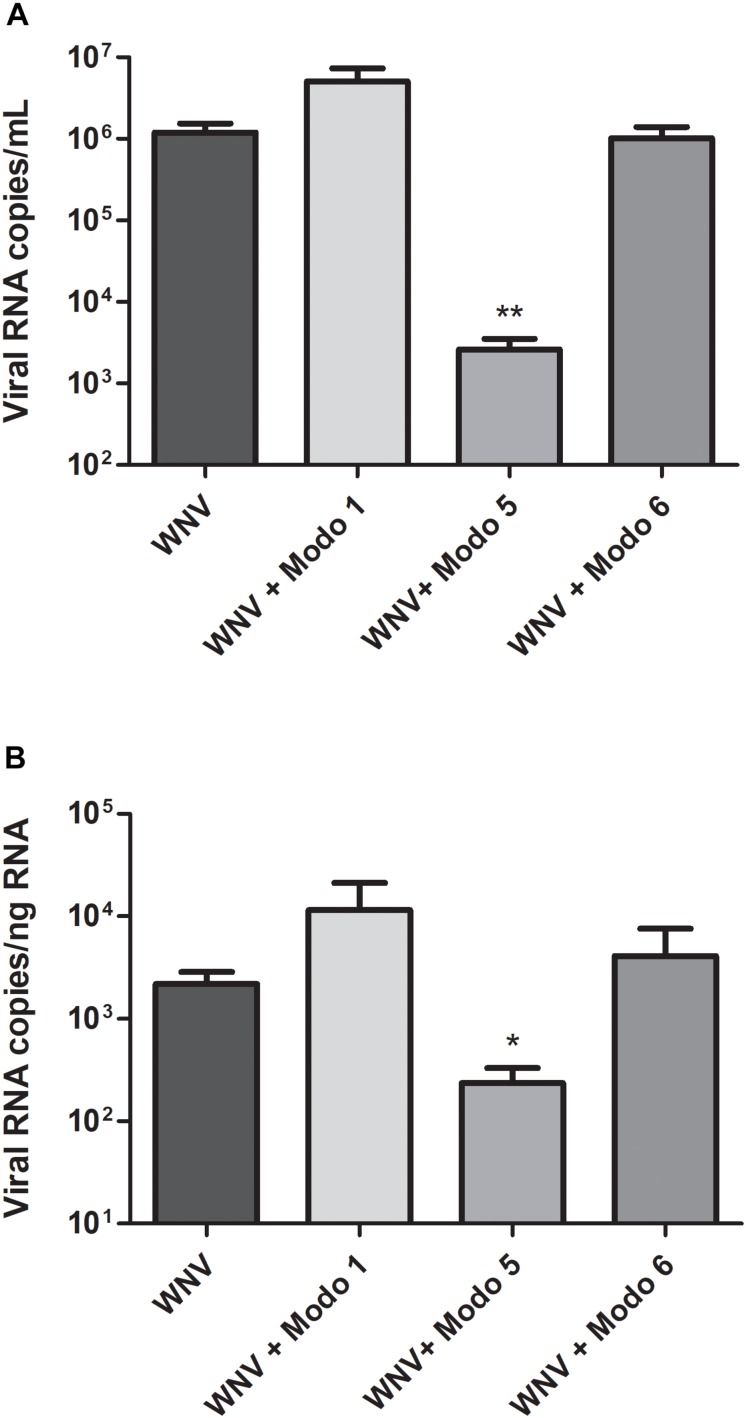
West Nile virus replication in human primary keratinocytes treated with ΔModoCath1, 5, and 6. Primary keratinocytes were treated with the peptides at a final concentration of 16 μg/mL before being infected with WNV at a MOI of 0.1 for 24 h. Viral loads were determined in the cell supernatant (**A**, in log10 viral RNA copies/mL) and in cell lysates (**B**, in log10 viral RNA copies/ng total RNA) from infected keratinocytes. Data are represented as mean ± SEM of three independent experiments. **p < 0.01, *p < 0.05, compared with the infected control without peptides. Modo 1, 5, and 6 indicate ΔModoCath1, 5, and 6, respectively.

Before studying the ability of ModoCaths to inhibit West Nile virus replication, their cytotoxic effects on human primary keratinocytes were evaluated (Table 3). The concentration of 16 μg/mL, resulting in cell viability greater than 90% for the three peptides, was chosen to test the antiviral activity of ΔModoCath1, 5, and 6. Results showed a potent inhibitory effect of ΔModoCath5 on WNV replication in primary keratinocytes; ModoCath1 and 6 exhibited no antiviral effects (Figure 4). The concentration of viral RNA was significantly reduced in supernatants of cells treated with ΔModoCath5, resulting in approximately 500-fold decrease in virus production. In the cell monolayer, ΔModoCath5 treatment also resulted in a 1-log (93%) decrease in WNV viral loads.

In the second step, the cell innate immune response to WNV infection was characterized in absence and presence of the peptide, in order to evaluate the potential immunomodulatory effects of ΔModoCath5 on cellular antiviral responses. The expression profile of players involved in the cellular antiviral response was studied by transcriptomic analyses. mRNA expression levels of molecules, such as the type III interferon interleukin 28A (IL28A), the chemokine CXCL10, as well as three interferon-stimulated genes (ISGs; IFIT2, ISG20, and viperin), which are known for their antiviral activities, were monitored (Supplementary Figure S7A). ΔModoCath5 treatment in keratinocytes during WNV infection did not significantly modulate the cellular antiviral response compared to that in untreated infected cells. The presence of ΔModoCath5 resulted in lower CXCL10, IFIT2, and IL28A mRNA levels, which can be related to the reduced viral replication in keratinocytes treated with this peptide. While viperin expression was not modified, ΔModoCath5 tended to increase ISG20 expression in WNV-infected keratinocytes. However, at the protein level, type III interferon secretion in response to WNV infection was not modulated by ΔModoCath5 treatment (Supplementary Figure S7B).

In addition, the virucidal properties of ΔModoCath1, 5, and 6 were assessed by measuring WNV infectious titers following pre-incubation in the presence of these peptides at a final concentration of 16 μg/mL for 1 h at 37°C. The infectious titer measured in the presence of the peptides was compared to that of the untreated virus suspension. No direct virucidal effect of the ModoCath peptides was observed (Supplementary Figure S8). Taken together, our results show that ΔModoCath5 strongly inhibits WNV replication in primary human keratinocytes by mechanism of action that remains to be defined.

## Discussion

Endogenous AMPs are natural substances encoded by a diverse group of genes in vertebrates to eliminate pathogens or control microbiota of the host. Although many AMPs are being discovered from diverse species, detailed analysis of their biological activity, which is critical for the development of possible clinical applications, is still lacking. Here, we sought to address this shortfall by characterizing the AMP repertoire of the gray short-tailed opossum by *in silico* analyses, particularly for cathelicidins, which are greatly expanded in the species in comparison to eutherian species (4, 13). We determined the core sequences of eight opossum cathelicidins predicted to have strong antimicrobial activity. Three selected peptides, ΔModoCath1, 5, and 6, were demonstrated to possess potent antimicrobial activity with different antimicrobial spectra, including potent antiviral activity against WNV.

The antimicrobial activity of a single opossum cathelicidin, ModoCath4, has been previously demonstrated (14). Determination of the active core sequence for cathelicidin propeptides depends on the predicted enzyme cleavage site, secondary structures, cationic charges, and hydrophobicity (3, 28, 29). In this study, we slightly revised the core sequence of three opossum cathelicidins previously thought to have no antimicrobial activity after adjusting the neutrophil elastase cleavage site (14). Interestingly, we were able to detect antimicrobial activity against pathogenic bacteria for all three tested peptides, suggesting that the precise determination of the active domain sequence is critical to assess the activity of cathelicidins. These results suggest that the five untested sequences listed in Table 1 may also show antimicrobial activities. Together with ModoCath4 from a previous study (14), antimicrobial activity has so far been confirmed for four opossum cathelicidins.

With the exception of certain marine organism AMPs that have been found to tolerate salt concentrations up to 450 mM, higher salt concentrations often interfere with AMP activity (43). Salt sensitivity is also observed in various antimicrobial peptides such as magainins, indolicidins, gramicidins, bactenecins (44). Notably, defensins have been reported to become inactivated in the presence of high salt concentrations (45, 46). In fact, studies have shown that lung infection by *P. aeruginosa* in cystic fibrosis patients is often related to inactivity of AMPs at the higher salt concentrations found in the lungs of these patients (46). For this reason, increasing the salt tolerance of AMPs to a minimum of 150 mM NaCl has been a goal of numerous studies (43, 47–49). Interestingly, the activity of ΔModoCath1, which had the highest net charge among the peptides used in this study, was potentiated in various salts at physiologic concentrations, while the bactericidal activity of ΔModoCath5 was negatively affected (Supplementary Table S7). Hence, the charge distribution and conformational stability appear to be important factors contributing to salt insensitivity (43, 47–49). Alternatively, the salt tolerance exhibited by ΔModoCath1 may have been attributed to the extreme positive net charge (+12) and structural stabilization by salts.

The antimicrobial properties of ΔModoCath1 and 5 were improved by a minimum 2-fold at solutions with a lower pH (Supplementary Table S7). This result is consistent with previous studies which reported that the bactericidal activity of AMPs is equal to or superior at lower pH than at a neutral pH (50). Particularly, His-rich peptides, such as clavanins which contain a number of histidines in place of the more common lysine or arginine residues, were potentiated at an acidic pH, reducing their microbicidal concentrations and shortening their killing times (51, 52). These enhanced bactericidal activities in acidic conditions is attributed to an increase in the positive charge of AMPs facilitating electrostatic interactions of peptides with anionic microbial surfaces. Our results suggest that ModoCaths, or their derivatives, may prove effective as treatment options for infections occurring in areas of the body with a physiologically acidic pH.

Therefore, the high level of antimicrobial activity elicited by ΔModoCath1 and 5 was due to the high net positive charge and amphiphilic α-helical conformation observed in CD spectroscopy (Figure 1). Although ΔModoCath1 and PMAP36 share only 42% sequence homology, they showed similar activity against *E. coli* (Figure 1D) which is likely due to the similar CD spectra for these two peptides upon LPS interactions, as well as their net charges (Table 1).

Further, ModoCath peptides induced blebs on bacterial surfaces similar to that observed following treatment with PMAP36 (Figures 3A,E,F). This phenomenon has been previously described for other AMPs with membrane-perturbing activity, such as magainin 2, temporin L and SMAP-29 (53–55). Additionally, the appearance of blebs has been reported as indicative of a given peptide’s ability to destabilize the outer membrane of gram-negative bacteria following displacement of divalent cations that function to bridge and neutralize LPS (56). The results from SEM analysis are consistent with those of the membrane disruption assay (Figure 2).

Comparison of AMP gene numbers among eutherian and marsupial species indicated the extensive expansion of cathelicidins in marsupials during evolution (Supplementary Table S9). Our analysis identified 19, 7, 11, and 11 cathelicidin genes from the gray short-tailed opossum (*Monodelphis domestica)*, tammar wallaby (*Macropus eugenii*), Tasmanian devil (*Sarcophilus harrisii*), and koala (*Phascolarctos cinereus*), respectively. This, in contrast to the presence of a single gene in humans and mice, suggests the importance of cathelicidins to marsupials, for instance to compensate for the lack of an adaptive immune system in neonate marsupials (8).

Using publicly available RNA-seq data (Supplementary Table S2), we detected the expression of eight cathelicidins–ModoCath1, 2, 4, 5, 7, 8, 16, and 18–from 5 tissues: placenta, lung, spleen, kidney, and Meckel’s cartilage and anterior malleus (Supplementary Table S10). ModoCath4 and 8 in particular were expressed at higher levels than the others. The diversity of cathelicidin expression was highest in the spleen.

Although antiviral properties of cathelicidins have been less intensively studied than their bactericidal effects, several cathelicidins including LL-37, protegrin-1, SMAP-29, BMAP-27, and frog temporin have shown antiviral activity against several pathogenic human viruses (15–18, 57). To our knowledge, our study is the first to report that ΔModoCath5 possesses strong antiviral activity against WNV. Although we only tested its antiviral activity on WNV, ΔModoCath5 may also exhibit broad antiviral activity against other flaviviruses and, potentially, other enveloped viruses. The antimicrobial spectrum of ModoCath5 seems to be similar to that of LL-37 showing both antibacterial and antiviral activities. LL-37 acts either through direct inactivation of the viral particles or upregulation of the cellular antiviral response (15, 16, 58, 59). However, by contrast with LL-37, ΔModoCath5 displayed no direct virucidal effect at the tested concentrations (Supplementary Figure S8). Moreover, ΔModoCath5 did not modulate inflammatory mediator production in WNV-infected human primary keratinocytes (Supplementary Figure S7). Therefore, further research is still required to determine the mechanism of antiviral activity of ΔModoCath5 assessed during keratinocyte infection.

Despite great interests in the therapeutic use of endogenous AMPs and current progresses on characterization of new AMPs, including research on its potency and specificity toward pathogens, action mechanisms, and cytotoxicity to mammalian cells, our knowledge is still limited. Animals such as opossums harboring a large-sized cathelicidin repertoire could become an interesting model to study the systematic effect of cathelicidins. Considering the broad-spectrum bactericidal and antiviral activity, ΔModoCath5 could be an interesting candidate for therapeutic exploitation.

## Data Availability Statement

The datasets generated for this study can be found in the GenBank database under the accession numbers: XM_001381586.3, XM_007476942.1, XM_007474477.1, XM_007499675.2, XM_007499676.2, XM_007499719.2, XM_007499718.2, XM_003341720.3, XM_007499716.1, XM_007500054.2, XM_007500053.2, XM_007500052.2, XM_007499717.2, XM_007499678.1, XM_007505482.2, XR_001623701.1, XM_007499715.1, XM_007499714.1, XM_007499677.1, XM_007491824.2, XM_001372004.3, XP_003339699.1, XP_007485040.1, XP_001381623.1, XP_007477004.1, XP_007474539.1, XP_007474439.1, XP_007474434.1, XP_007474435.1, XP_007474436.1, XP_007474534.1, XP_007474533.1, XP_007474532.1, XP_007474437.1, XP_016283821.1, XP_007503439.1, XP_016281316.1, XP_007474984.1, XP_016281317.1, XP_016284649.1, XP_001381804.1, XP_007474950.1, XP_007474951.1, XP_007474952.1, XP_001381797.1, XP_007474444.1, XP_007474443.1, XP_007475462.1, XP_007475432.1, XP_016287018.1, XP_001374266.1, XP_016287021.1, XP_007475429.1, XP_016288248.1, XP_001372041.1, XP_007491886.1, XP_007499778.1, XP_007499740.1, XP_007499737.1, XP_007499738.1, XP_007499781.1, XP_003341768.1, XP_007499780.1, XP_007500114.1, XP_007500115.1, XP_007500116.1, XP_007499779.1, XP_007505544.1, XP_007499777.1, XP_007499739.1, XP_007499776.1, and XP_007505545.1. The raw data supporting the conclusions of this article will be available in Supplementary Data by the authors.

## Ethics Statement

The studies involving human participants were reviewed and approved by the Ethics Committees of Poitiers Hospital and Konkuk University Hospital. The patients/participants provided their written informed consent to participate in this study.

## Author Contributions

HC, CP, and CB designed and coordinated the study. HC, JY, AL, NL, QL, NS, and HJ performed all experiments to characterize ModoCaths. HC, CB, and CP analyzed the data and interpreted the results. JY and BA carried out bioinformatic analysis. HC, JY, CB, and CP wrote the manuscript. KH and J-HK provided administrative, technical, or material support. The final version of the manuscript was reviewed by all the authors.

## Conflict of Interest

The authors declare competing financial interests – the peptide sequences ΔModoCath1, 5, and 6 are the subject of domestic and foreign patent applications by Konkuk University. The remaining authors declare that the research was conducted in the absence of any commercial or financial relationships that could be construed as a potential conflict of interest.

## Footnotes

1 http://www.uniprot.org/uniprot/

2 http://blast.ncbi.nlm.nih.gov

3 https://www.ncbi.nlm.nih.gov/sutils/splign

4 http://www.cbs.dtu.dk/services/SignalP/

5 https://www.ebi.ac.uk/Tools/hmmer/

6 https://dbaasp.org/prediction

7 http://tcoffee.crg.cat/apps/ampa/do

8 http://bioinf.cs.ucl.ac.uk/psipred/

9 http://web.expasy.org/peptide_cutter/

10 https://prosper.erc.monash.edu.au

11 http://aps.unmc.edu/AP/prediction/prediction_main.php

12 https://web.expasy.org/protparam/

13 https://www.ncbi.nlm.nih.gov/sra
