# Supplementary material for: Opossum Cathelicidins Exhibit Antimicrobial Activity Against a Broad Spectrum of Pathogens Including West Nile Virus
Source: Front Immunol. 2020 Mar 3;11:347. doi: 10.3389/fimmu.2020.00347 (PMC7063992; doi:10.3389/fimmu.2020.00347)
Supplement: Supplementary file 1 [file Data_Sheet_1.docx]

Supplementary Material

**Supplementary Figures**


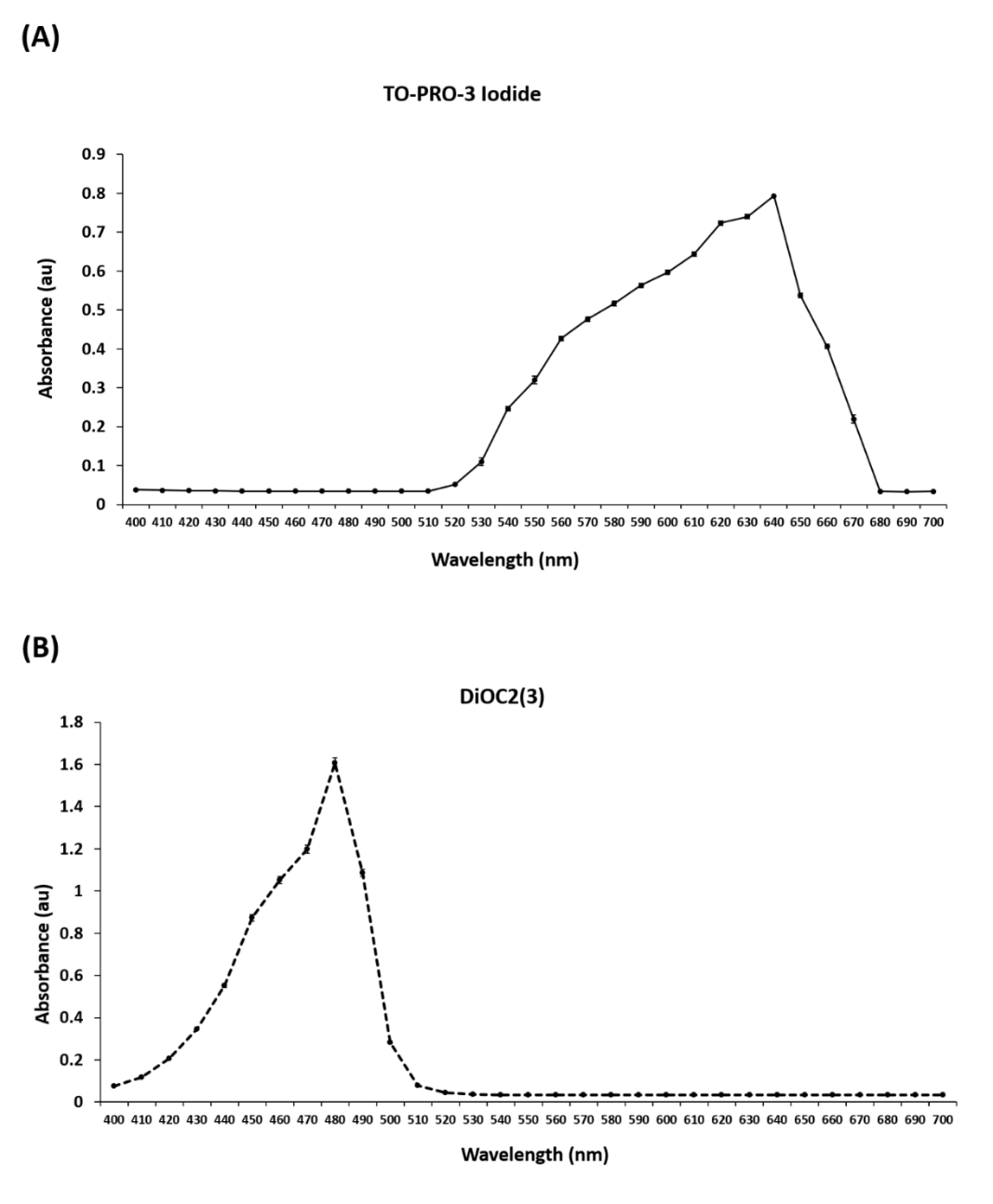


**Figure S1.** Confirmation of absorbance spectra for TO-PRO-3 iodide and DiOC_2_(3). To determine the optimal UV wavelength of excitation for two fluorescence dyes, absorbance spectra at 400-700 nm with 10 nm interval were obtained. The maximal absorbance for TO-PRO-3 **(A)** and DiOC_2_(3) **(B)** was detected at 640 nm and 480 nm, respectively.


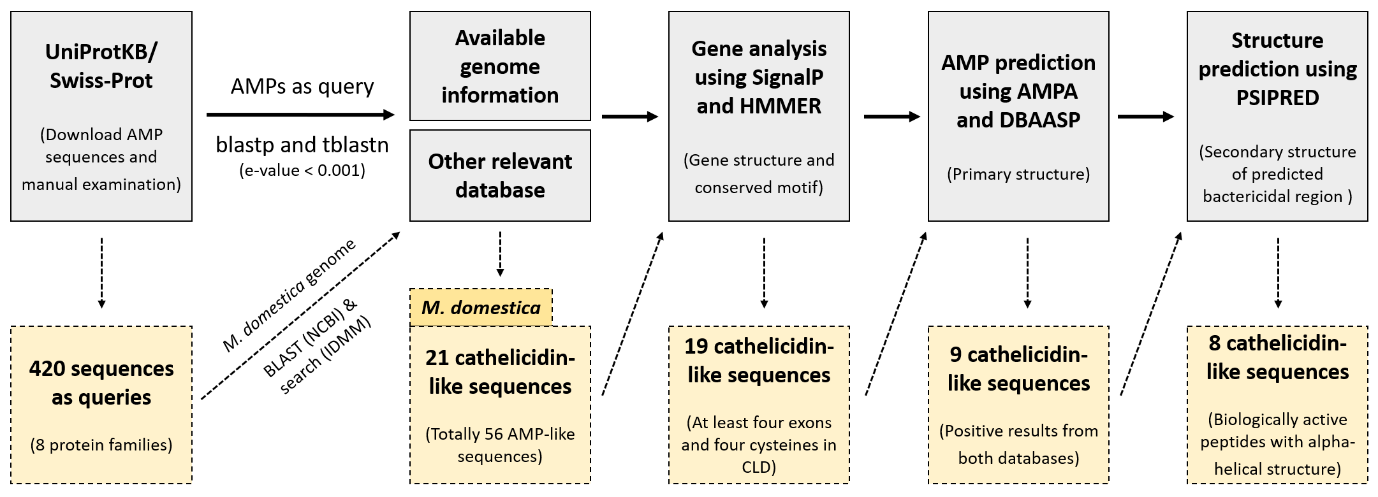


**Figure S2.** Strategy used in the in-silico genome analysis to identify and characterize AMPs D**o**wnloaded AMP-related sequences from UniProtKB/Swiss-Prot database, 420 representative sequences comprising 8 subfamilies with antimicrobial activity were blasted against the genome of *M. domestica.* A total of 21 cathelicidin-like proteins were obtained by performing the blast together with searches against NCBI and IDMM databases. The putative cathelicidin-like sequences were further analyzed using SignalP, HMMER, AMPA, DBAASP, and PSIPRED. Finally, eight cathelicidin peptides with predicted antimicrobial domain and functional structure were selected as promising candidates for having antimicrobial activity.


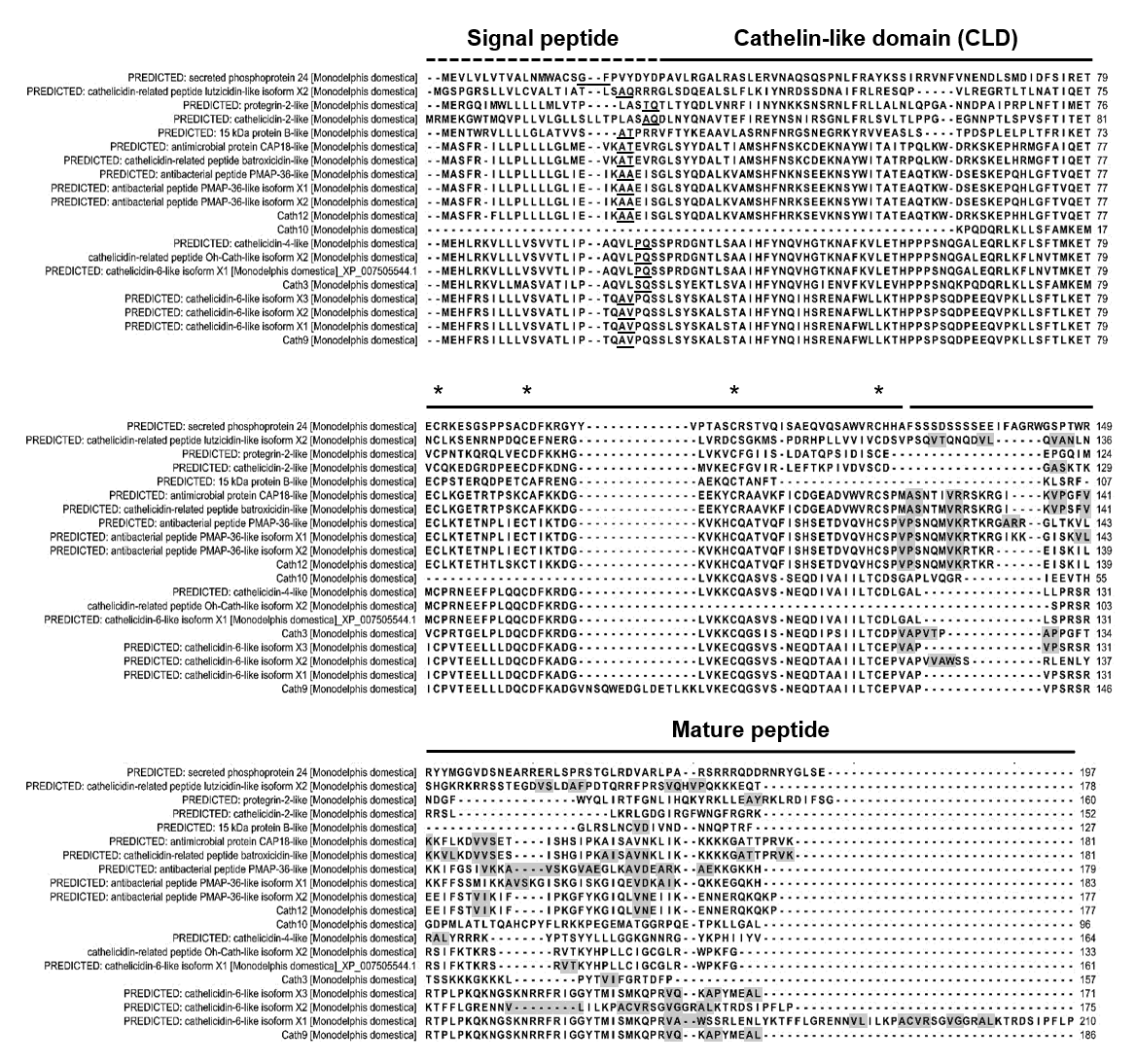


**Figure S3.** Comparison of cathelicidin-like sequences in the genome of *Monodelphis domestica*. The 20 cathelicidin-like sequences from BLAST, NCBI, and IDMM analyses were compared by multiple sequence alignment using MUSCLE. Gap and conserved four cysteines in cathelin-like domain (CLD) are indicated by dashes (-) and an asterisk (*), respectively. The signal peptide cleavage sites are underlined, and the signal peptide region is indicated by a dotted line above the aligned sequences. The predicted proteolytic cleavage sites with neutrophil elastase to form mature cathelicidins are indicated by gray highlights. The CLD sequence of first four predicted cathelicidin sequences differed from the rest of the sequences. The accession number for each sequence is indicated in Table S5.


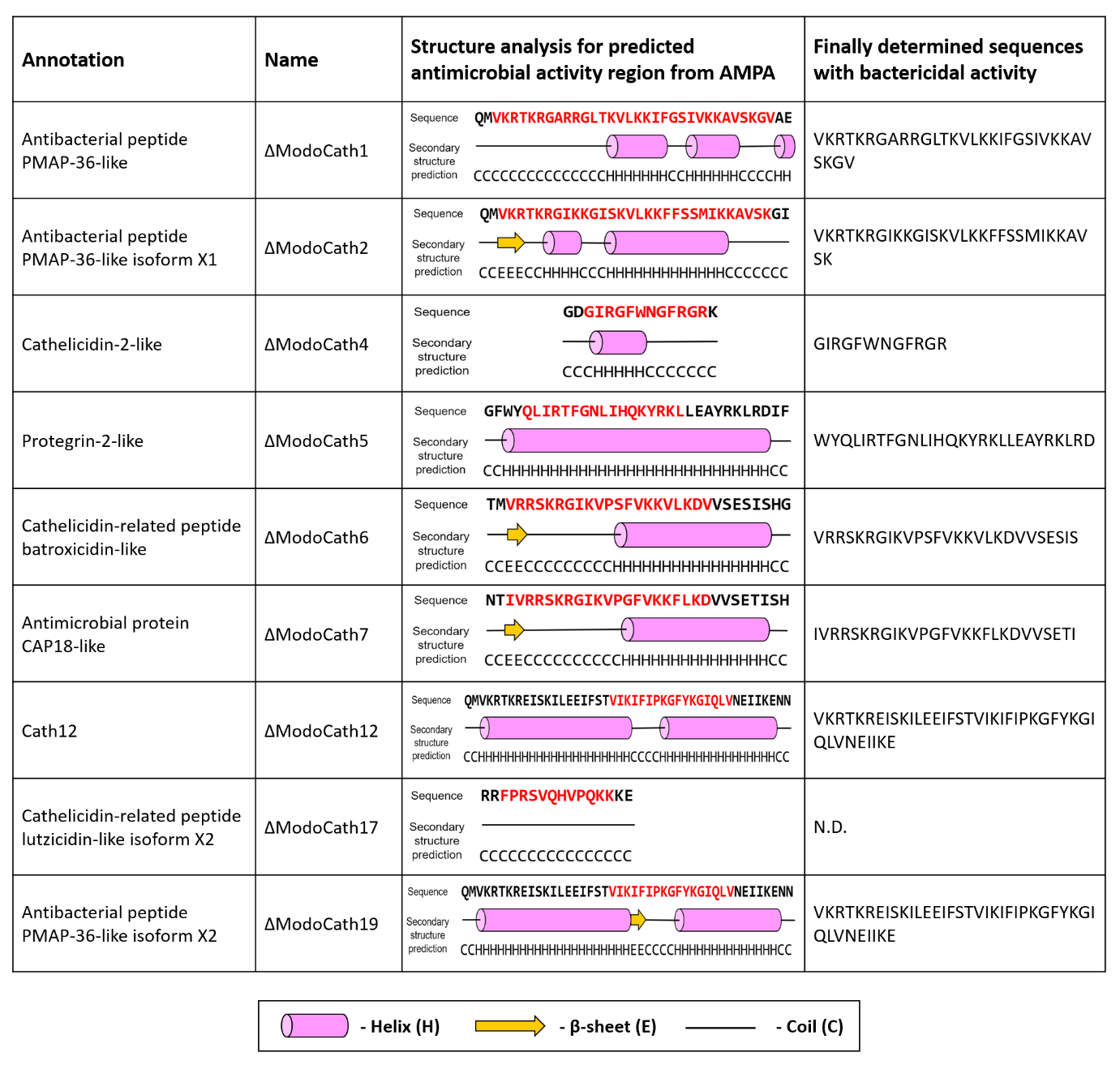


**Figure S4.** Results from AMPA and PSIPRED analyses for prediction of the antimicrobial activity core region and secondary structures. The sequences are indicated in red as predicted antimicrobial domains resulting from AMPA analysis. The cylinders, arrows, and lines of different colors represent alpha-helix, beta-sheet, and coil, respectively.


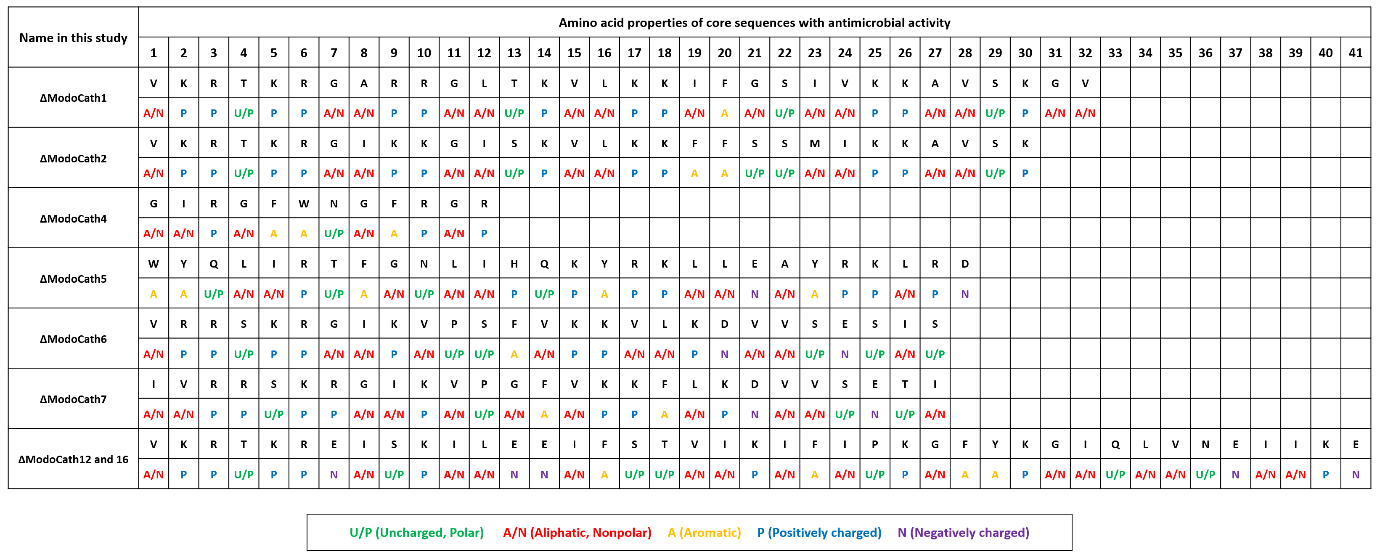


**Figure S5.** Comparison of amino acid properties of the predicted core sequences with antimicrobial activity among eight cathelicidins. A high similarity of amino acid properties was observed between ΔModoCath1 and 5 and between ΔModoCath6 and 7.


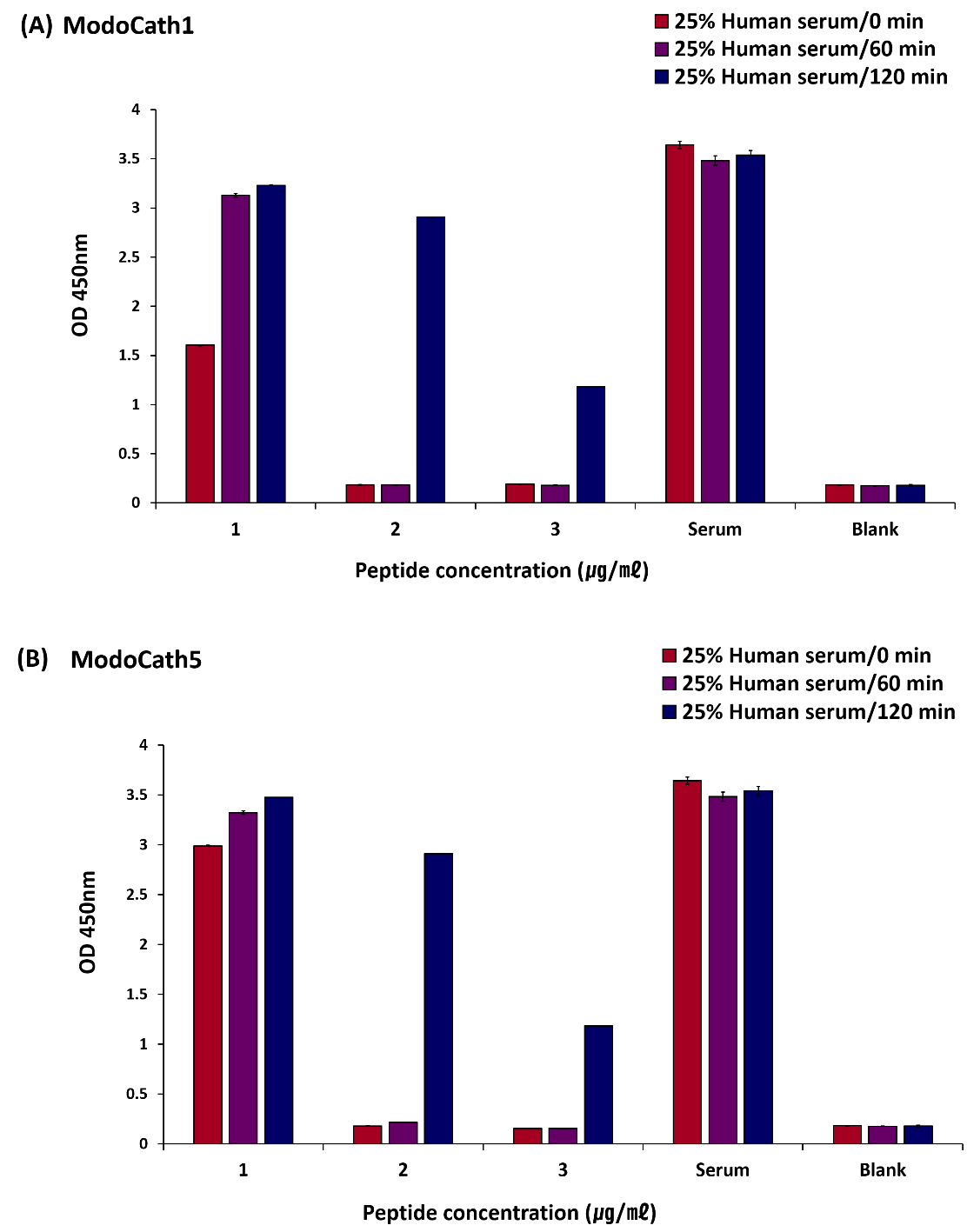


**Figure S6.** Evaluation of antimicrobial activity of ΔModoCath1 and 5 after incubation with serum. ΔModoCath1 and 5 were incubated with 25% pooled human serum each for 0, 60 and 120 min, and their antimicrobial activities against *E. coli* were determined by measuring the absorbance (OD) at 450 nm. The X and Y axes indicate peptide concentration and absorbance at 450 nm, respectively. Samples containing serum in the absence of the peptides served as negative controls with intact bacteria growth. The antimicrobial activity of **(A)** ΔModoCath1 and **(B)** ΔModoCath5 at 1x MIC was highly affected by incubation with serum; however, the activity at a higher concentration was considerably stable.


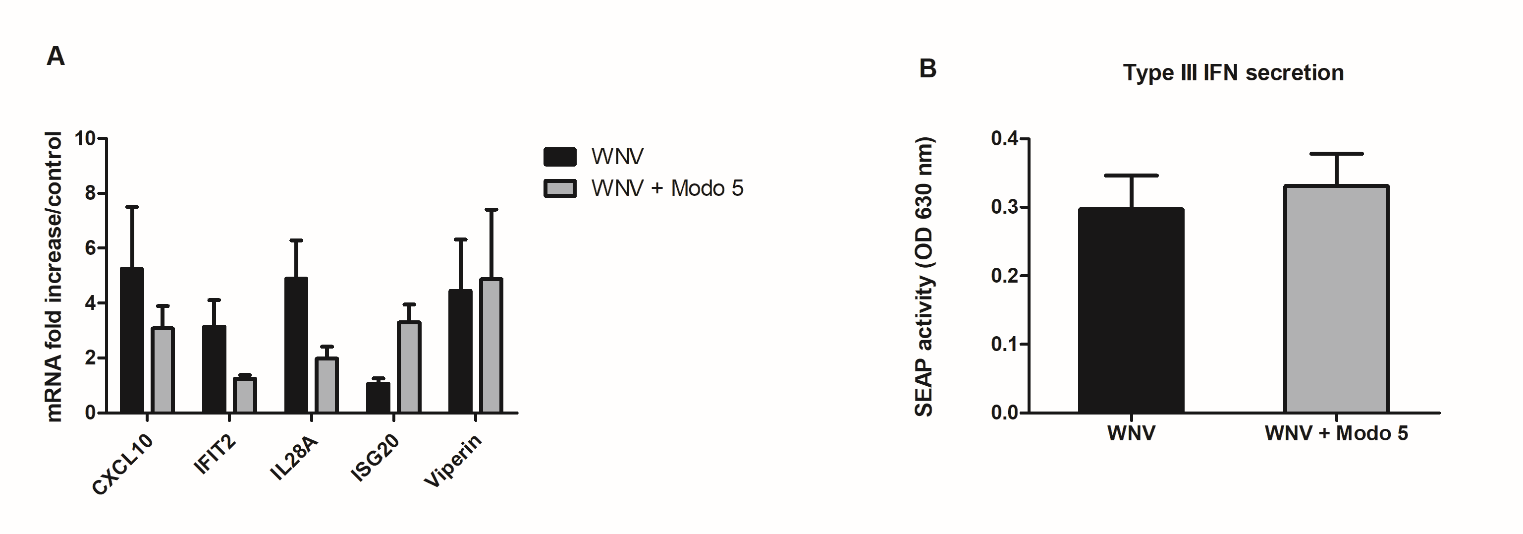


**Figure S7.** Modulation of the WNV-induced anti-viral response of human primary keratinocytes by ΔModoCath5. (A) CXCL10, IFIT2, IL28A, ISG20, and viperin mRNA expression was quantified in human primary keratinocytes infected with WNV for 24 h at MOI of 0.1 in the presence or absence of ΔModoCath5 at a final concentration of 16 µg/mL. mRNA expression levels are expressed as the fold increase above uninfected cultures. No significant difference was observed between the two groups using Mann-Whitney test (p > 0.05). (B) Type III interferon (IFN) secretion levels of human primary keratinocytes infected with WNV for 24 h at MOI of 0.1 in the presence or absence of ΔModoCath5 at a final concentration of 16 µg/mL. Quantification of active type III IFNs was performed using HEK-Blue™ reporter cells and activity of secreted embryonic alkaline phosphatase (SEAP) was determined by measuring the optical density (OD) at 630 nm. Data are presented as mean ± SEM of at least three independent experiments. Modo 5 indicates ΔModoCath5.


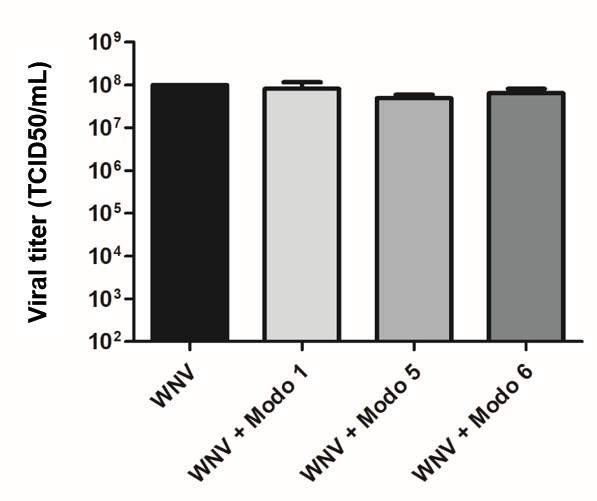


**Figure S8.** Evaluation of virucidal properties of ΔModoCath1, 5, and 6 on WNV. WNV suspension was pre-incubated in the absence or presence of ΔModoCath1, 5, and 6 at a final concentration of 16 µg/mL for 1 h at 37°C before titration by the end-point dilution assay on Vero cells. Viral titers are expressed as TCID50/mL. Data are presented as mean ± SEM of three independent experiments. Modo 1, 5, and 6 indicate ΔModoCath1, 5, and 6, respectively.
